# Excellent clinical and radiological outcomes after both open flake refixation and autologous chondrocyte implantation following acute patella dislocation and concomitant flake fractures

**DOI:** 10.1007/s00167-022-06899-3

**Published:** 2022-02-26

**Authors:** Yannick J. Ehmann, Lea Zuche, Andreas Schmitt, Daniel P. Berthold, Marco-Christopher Rupp, Lukas N. Muench, Alexander Otto, Klaus Woertler, Andreas B. Imhoff, Julian Mehl

**Affiliations:** 1 Department of Orthopedic Sports Medicine, Technical University of Munich, Ismaninger Str. 22, 81675 Munich, Germany; 2 Department of Trauma, Orthopaedic, Plastic and Hand Surgery, University Hospital of Augsburg, Augsburg, Germany; 3 OFZ Weilheim, Weilheim, Germany; 4 Department of Radiology, Technical University of Munich, Munich, Germany

**Keywords:** Flake fracture, Refixation, ACI, Patellofemoral instability, Autologous chondral implantation

## Abstract

**Purpose:**

To investigate clinical and magnetic resonance (MR) imaging results of patients undergoing patella stabilization with either open flake refixation (oFR) or autologous chondrocyte implantation (ACI) and concomitant soft tissue patella stabilization after sustaining primary, acute patella dislocation with confirmed chondral and/or osteochondral flake fractures. It was hypothesized that refixation will lead to better results than ACI at mid-term follow-up.

**Methods:**

A retrospective chart review was conducted to identify all patients undergoing oFR or ACI after sustaining (osteo-)chondral flake fractures and concomitant soft tissue patella stabilization following primary, acute patella dislocation between 01/2012 and 09/2018 at the author’s institution. Patients were excluded if they were aged < 14 years or > 30 and had previous knee surgeries at the index knee. Clinical outcomes were assessed using the Tegner activity score, Kujala score, subjective IKDC score, and the KOOS score at a minimum follow-up of 24 months postoperatively. MR images were assessed using the Magnetic Resonance Observation of Cartilage Repair Tissue (MOCART) 2.0 knee score. Thirty patients were included in the study, with 16 patients assorted to the oFR group and 14 patients to the ACI group (Follow-up 81%).

**Results:**

Demographic data did not show significant group differences (oFR: 6 females, 10 males; age 26.9 ± 5.6 years, FU: 57 months (27–97 months); ACI: 9 females, 5 males; age 25.5 ± 4.9 years, FU: 51 months (29–91 months); n.s.). Defect location was similar in both groups (oFR: 12 × patella/4 × lateral femoral condyle; ACI: 12/2; n.s.). Both groups showed excellent clinical outcomes, with no statistically significant difference between both the groups (oFR group vs. ACI group: Tegner: 5.1 ± 1.8 vs. 5.1 ± 1.4; Kujala: 86.1 ± 12.6 vs. 84.9 ± 9.1; IKDC: 83.8 ± 15.0 vs. 83.6 ± 11.3; KOOS: 83.3 ± 14.0 vs. 83.6 ± 12.0; n.s.). One patient in each group suffered a patella re-dislocation and needed revision surgery. The MOCART 2.0 score showed good results for the oFR group (68.2 ± 11.1) and the ACI group (61.1 ± 16.9) while no significant differences were noted between both the groups. The inter-rater reliability was excellent (0.847).

**Conclusion:**

Open refixation of (osteo-)chondral fragments in patients after sustaining acute patella dislocation with (osteo)-chondral flake fractures led to good clinical and radiological results at a minimum follow of 24 months, showing that it is a good surgical option in the treatment algorithm. However, if open refixation is not possible, ACI may be an excellent fallback option in these younger patients with equally good clinical and radiological outcomes, but requiring a second minimally invasive surgery.

**Level of evidence:**

III.

**Supplementary Information:**

The online version contains supplementary material available at 10.1007/s00167-022-06899-3.

## Introduction

Patella dislocations account for approximately 2–3% of all knee injuries. They are highly associated with patellofemoral cartilage defects and the risk of (osteo)-chondral flake fractures are reported in up to 58% of patients [11, 17, 27]. Due to the limited regenerative capacity of articular cartilage and the associated increased risk of developing osteoarthritis, rapid diagnosis and appropriate therapy are crucial [4, 30].

In case of flake fractures, the primary goal is to refix the sheared articular cartilage while the potential for successful healing decreases over time. Various techniques are described in current literature, although a lack of consensus exists showing superiority for a specific technique [15]. However, refixation of the sheared-off fragment may only be successful, if the fragment is intact and the surrounding cartilage enables good embedding. Otherwise, if refixation is not indicated or fails, autologous chondrocyte implantation (ACI) is an established alternative, especially in younger patients with good healing potential. Subsequently, the efficacy of ACI has been proved by a large number of high-quality studies, [2, 29] although earlier publications described less predictable results for patellofemoral defects in comparison with other locations [23]. More recent studies did not confirm these findings provided that accompanying pathologies such as instabilities of the patella were treated [7, 10, 21, 32]. While those two techniques represent the most commonly used ones other techniques like allografts and scaffolds can be used as well. Regardless of which surgical technique is used for the repair of patellofemoral flake fractures, additional patella stabilization may be necessary to reduce the risk of reluxation and to protect the repaired cartilage [1, 13].

A previous systematic literature review including 19 studies published between 1964 and 2011 investigated the results after treatment of (osteo-)chondral flake fractures in the knee joint [15]. However, only case series without controls were reported and additional patella stabilizing procedures were not considered. In a more recent comparative study, Gesslein et al. investigated the clinical outcome following the refixation of (osteo-)chondral lesions after patella dislocation and demonstrated superior results in comparison with debridement of the defect zone [6]. However, no comparison with alternative repair techniques and no radiological examinations to evaluate the repaired tissue were performed.

The purpose of this study was to investigate clinical and radiological outcomes using MR imaging in patients undergoing open flake refixation or ACI with additional soft tissue stabilization after sustaining acute patella dislocation with (osteo)-chondral flake fractures. It was hypothesized that flake refixation will lead to better clinical and radiological outcomes when compared to ACI at a minimum follow-up of 24 months.

## Materials and methods

Following Institutional Review Board approval of the Technical University of Munich (IRB 196/17S**)**, a retrospective chart review was conducted to identify all patients undergoing open flake refixation or ACI after sustaining (osteo-)chondral flake fractures and concomitant soft tissue patella stabilization following a primary, acute patella dislocation between 01/2012 and 09/2018 at the author’s institution. Patients were included if they had confirmed retropatellar/femoral (osteo-)chondral flake fractures after sustaining primary patella dislocation, and if they were treated using either flake refixation or ACI with additional soft tissue stabilization. Patients were excluded if they were aged < 14 years or > 30 years; if they had previous knee surgeries at the index knee; or if they had any additional concomitant procedures other than flake refixation/ACI or soft tissue stabilization.

Based on the surgical procedure, all patients were allocated to one of two groups; (1) primary open flake refixation of the sheared (osteo-)chondral fragment (“oFR”); (2) autologous chondral implantation (“ACI”). The minimum follow-up was 24 months.

### Surgical technique

**Fig. 1:**
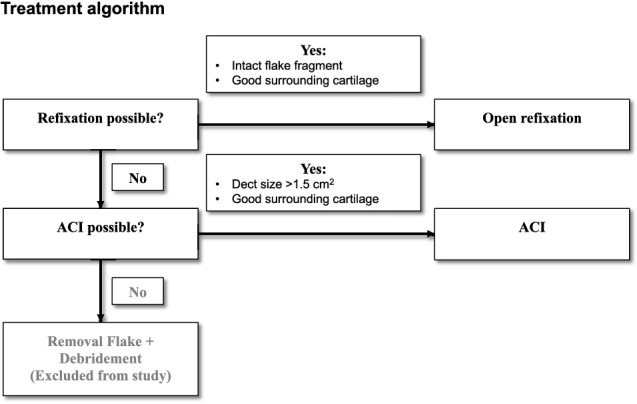
Treatment algorithm used in this study to determine which surgical procedure was used

All included patients had one of the following surgeries: primary open flake refixation of the (osteo-)chondral fragment (“oFR”) or third-generation autologous chondrocyte implantation (“ACI”). Primary refixation was performed in all patients with fragments eligible for refixation. Eligibility was checked at primary arthroscopy and defined as an intact sheared-off fragment with good surrounding cartilage at the defect zone that enables good embedding. Sometimes the sheared-off fragment does not fit in the defect zone. In this case, it can be helpful to cut and adjust the fragment with a scalpel to create a fitting form. If refixation was not possible, indication for third-generation ACI was checked and performed for all defects > 1.5 cm^2^ on the patella and lateral femoral condyle with surrounding healthy cartilage. Smaller defects and defects on the edge of the chrondal zone were treated with removal of the flake and the patients were not included in this study (Fig. 1).

**Fig. 2:**
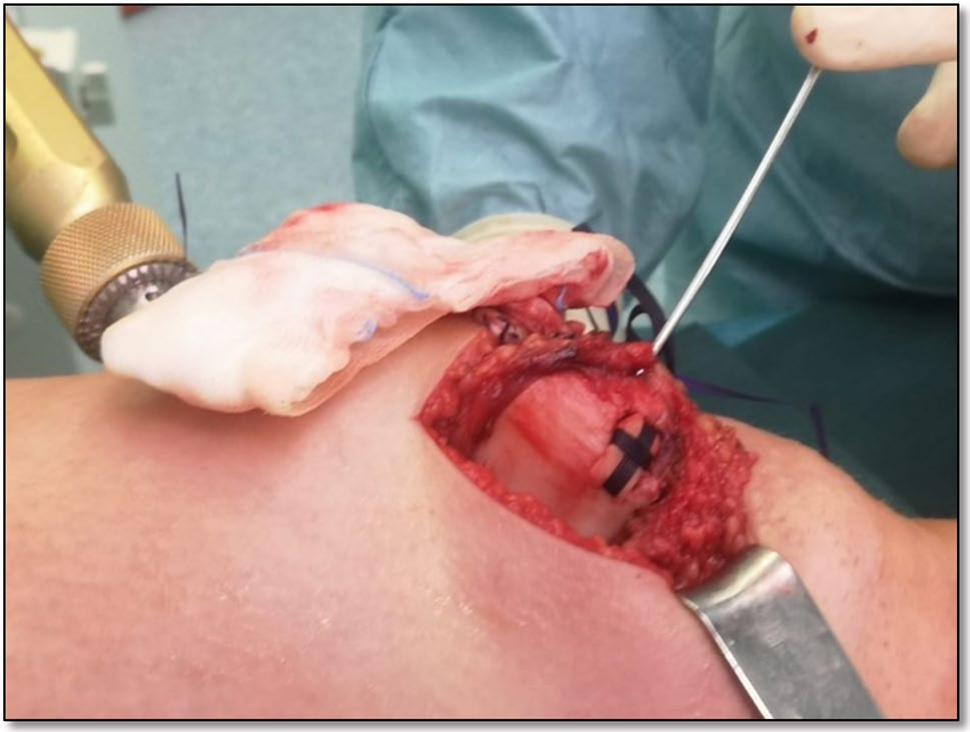
Open refixation of a retropatellar flake fracture using bioabsorbable tapes

In the event of retropatellar flake refixation, a medial parapatellar arthrotomy was performed, with patella everted. For defects at the lateral femoral condyle, a lateral mini-arthrotomy was performed [33]. For refixation of fragments, bioabsorbable tapes or mini-screws were used [6, 15] (Fig. 2). The bioabsorbable vicryl tapes are fixated in the patella with Fibertak anchors (Arthrex, Naples, USA) in a cross formation to hold the fragment in place. The mini-screws are used when the fracture is located on the lateral femoral condyle and it is not possible to position the bioabsorbable tapes around the fracture to fixate it. ACI was performed in two surgical steps. During primary arthroscopy, healthy cartilage specimens were harvested and sent to a laboratory (TETEC Tissue Engineering Technologies AG, Reutlingen, Germany). The chondrocytes were then isolated and proliferated in vitro for 3–5 weeks before they were implanted into the defect zone in a second surgical procedure [25]. All patients suffered from patellofemoral instability and were treated with either reconstruction of the medial patellofemoral ligament (MPFL) using the gracilis tendon or refixation of the medial retinaculum (in younger patients with open growth plates) [16]. If flake refixation was performed, patella stabilization was conducted in the same surgery. In the case of ACI, patella stabilization was performed in the second surgery together with the implantation of the chondrocytes.

### Postoperative rehabilitation

Each patient underwent a structured rehabilitation protocol for a minimum of 3 months postoperatively. Patients had to wear a brace with limited range of motion (ROM) for 6 weeks. Weight-bearing was limited to 20 kg for 6 weeks. Rehabilitation began on the first postoperative day under the direction of a trained physical therapist. After flake refixation, passive ROM was limited to extension/flexion 0/0/90° for 3 weeks and active ROM was limited to extension/flexion 0/0/90° for 6 weeks. Following ACI, passive and active ROM was limited to extension/flexion 0/0/45° for 3 weeks and to 0/0/90° for additional 3 weeks. Full pivoting sporting activities were not allowed for 6–12 months, contact sporting activities were not allowed for 9–12 months.

### Clinical outcome

Clinical outcomes were assessed using previously validated outcome scores. The Tegner Score was used to evaluate the work and sporting activities of the patients [8, 18]. The Kujala Score was used to measure the anterior knee pain and the associated problems of the knee joint [5]. The International Knee Documentation Committee (IKDC) subjective score was used to measure the symptoms, function, and sporting activity of the knee joint [12]. The “Knee Osteoarthritis Outcome Score” (KOOS) and its subscores were used to measure the impairment of the knee joint regarding pain, symptoms, activities of daily living, sporting activities, and quality of life [24]. Previous studies have confirmed these scores in terms of reliability, validity, and responsiveness.

Revision surgery, reluxation of the patella, postoperative complications, and deficits in range of motion were described and defined as a failure.

### Radiological outcome

**Fig. 3:**
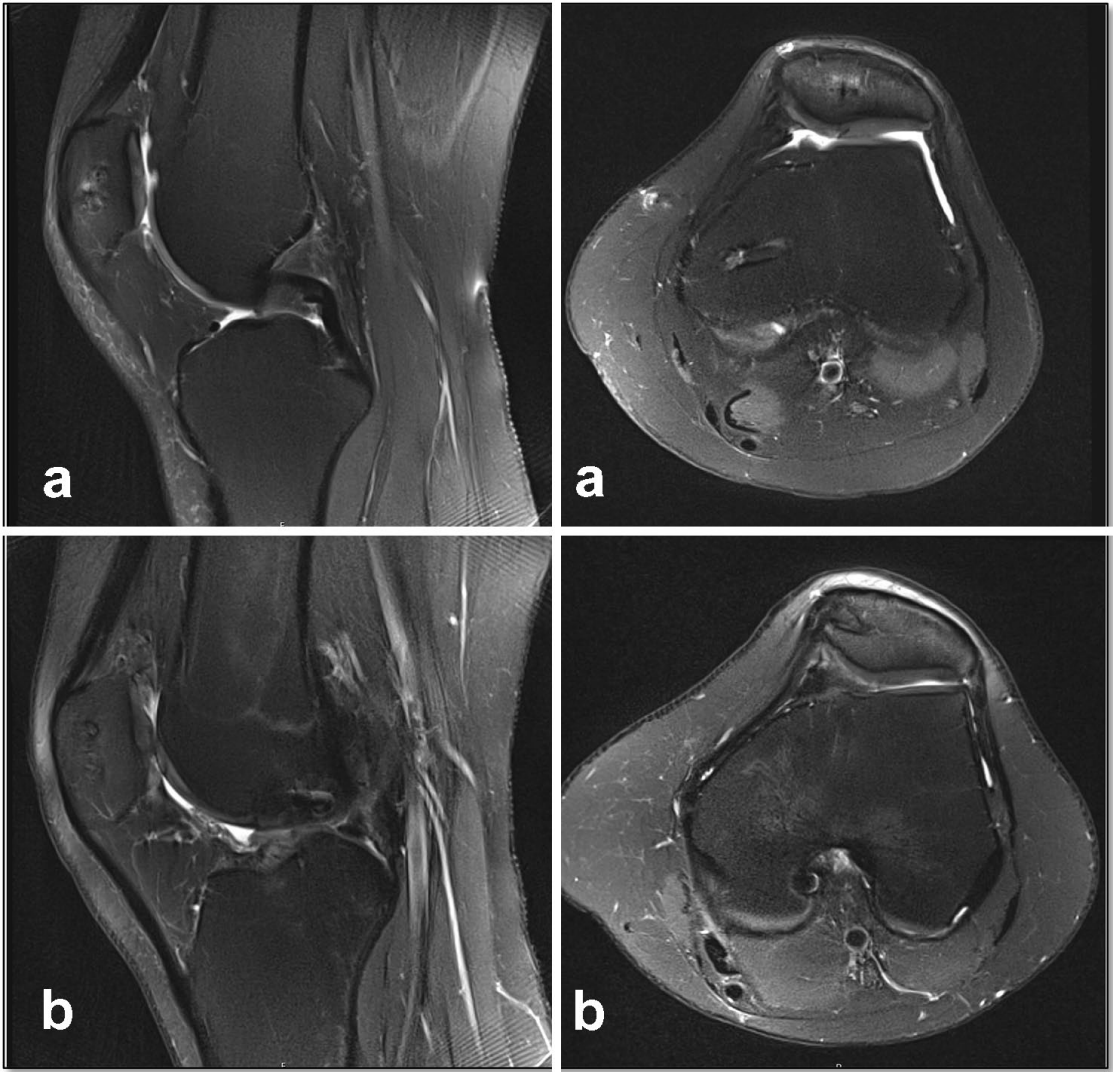
MR images at follow-up after open refixation (**a**) and ACI (**b**) show results after surgery with good integration of chondral tissue. Images after refixation (**a**) show good integration of the flake fragment after completed healing reaction. Images after ACI (**b**) show a good integration of the transplanted chondral tissue, with minor hypertrophy

MR imaging of the affected knee joint was performed at follow-up together with the clinical follow-up, to assess the repaired cartilage using the “Magnetic Resonance Observation of Cartilage Repair Tissue (MOCART) 2.0 knee score” [26]. All MR examinations were performed on the same 3.0-T whole-body MR scanner (Siemens, Erlangen, Germany) with the use of a dedicated knee coil. Sagittal and axial intermediate-weighted TSE sequences with spectral fat saturation were obtained to evaluate the articular cartilage (Fig. 3).

### Statistical analysis

Statistical analysis was conducted using Stata statistical software (StataCorp. 2017. Stata Statistical Software: Release 15. College Station, TX: StataCorp LLC). Normal distribution of quantitative variables was examined and graphically confirmed with the Shapiro–Wilk normality test. Normally distributed data were represented as mean ± standard deviation (SD) and not normally distributed data were represented as median and 25–75% interquartile range (IQR). Qualitative variables were represented as absolute and relative frequencies. The demographic data and the clinical outcome scores were compared between the “oFR”- and “ACI-” group. Quantitative data were compared using the t-test for normally distributed data and the Mann–Whitney-*U*-test for not normally distributed data. Between-group comparisons of qualitative data were performed using the Chi-square test and Fischer’s exact test. Interrater-reliability for the MOCART 2.0 score was evaluated using the interclass correlation coefficient (ICC). The alpha level for all analyses was set at 5%. A post hoc power analysis was conducted for comparison of the clinical results using a 2-sided test. Consequently, it was shown that the sample size of 30 in this study could achieve an adequate power of 0.90 with an *α* of 0.05 for the Kujala Score. The sample size calculation and the power analysis were performed using G*power 3.1.

## Results

**Fig. 4:**
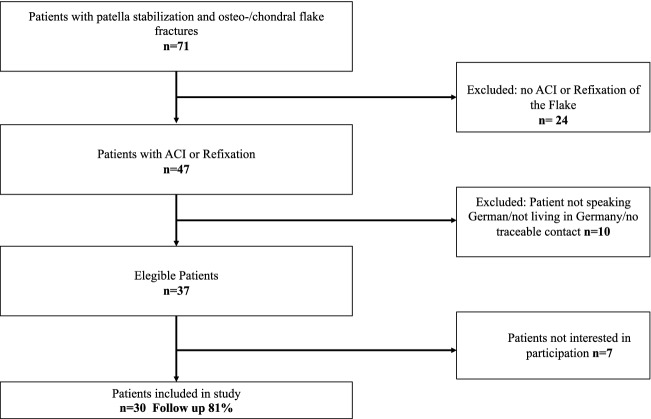
Flowchart displaying patient selection process

The final study cohort comprised of 30 patients (Fig. 4). Flake refixation was performed in 16 patients and ACI was performed in 14 patients. There were no significant differences regarding the demographic data, defect location, defect size, or follow-up time (Table 1). The median time between luxation and refixation of the fracture was 7 days (min: 2 days; max: 17 days). Two patients in the oFR group were treated with screw fixation with metal screws.

**Table 1:** Demographic data of the patient cohort

		Refixation		ACI		Total		*p* value
Gender		10 (62%)	6 (38%)	5 (36%)	9 (64%)	15 (50%)	15 (50%)	n.s.
Male	Female							
Age at follow-up (y)		26.9 ± 5.6		25.5 ± 4.9		26.4 ± 5.2		n.s
BMI (kg/m^2^)		24.0 ± 2.7		22.0 ± 2.6		23.1 ± 2.8		n.s.
Side		9 (56%)	7 (43%)	6 (43%)	8 (57%)	15 (50%)	15 (50%)	n.s.
Right	Left							
Localization		12 (75%)	4 (25%)	13 (87%)	2 (13%)	25 (81%)	6 (19%)	n.s.
Patella	LFC							
Size of defect (cm^2^)		2.6 ± 1.6		3.3 ± 1.6		2.9 ± 1.6		n.s.
Concomitant procedure		13 (82%)	3 (18%)	13(93%)	1 (7%)	26 (87%)	4 (13%)	n.s.
MPFL	RR							
Follow-up (months)		57.0 ± 16.4 (min: 27–max: 97)		51.1 ± 17.6 (min: 29–max: 91)		54.2 ± 17.0 (min: 27–max: 97)		n.s.

*n* number; *LFC* lateral 7 femoral condyle; *MPFL* medial patellofemoral ligament reconstruction; *RR* retinaculum refixation; *y* years; *n.s.* non-significant; *BMI* body mass index

### Clinical outcome

**Fig. 5:**
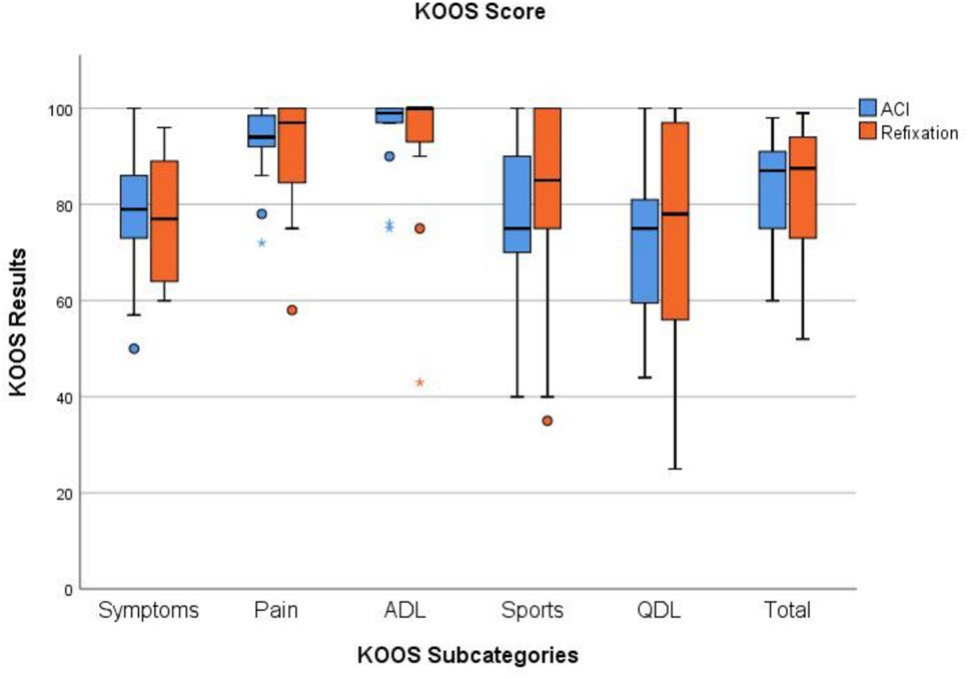
Results of the KOOS. The box plots show the median, the IQR and the range of the results in group comparison for each subcategory and the total score. Abbreviation: *ADL* activities of daily life; *QDL* quality of daily life

Both groups showed excellent postoperative clinical outcome scores at a minimum follow-up of 24 months with no significant differences between both groups (Table 2, Fig. 5). Only one patient from the oFR group sustained a traumatic patella re-dislocation, which was treated using a MPFL-re-reconstruction. Similarly, one patient from the ACI group had a new traumatic reluxation of the patella which did not require further surgical intervention. 73% of patients were able to return to their previous level of sports (oFR: 75%; ACI: 71%; n.s.). In the clinical examination, none of the patients had a flexion deficit > 10°.

**Table 2:** Clinical outcome scores of the patient cohort

		Refixation		ACI		Total		*p* value
VAS		0.6 ± 1.8		1.0 ± 1.3		0.8 ± 1.5		n.s.
Tegner		5.1 ± 1.8		5.1 ± 1.4		5.1 ± 1.6		n.s.
Kujala		86 ± 13		85 ± 9		86 ± 11		n.s.
KOOS symptoms		77 ± 13		78 ± 15		78 ± 14		n.s.
KOOS pain		91 ± 13		93 ± 9		92 ± 11		n.s
KOOS ADL		93 ± 15		95 ± 9		94 ± 12		n.s.
KOOS sports		81 ± 22		76 ± 19		79 ± 20		n.s.
KOOS QDL		74 ± 25		70 ± 18		72 ± 21		n.s.
KOOS total		83 ± 14		83 ± 12		83 ± 13		n.s.
IKDC 2000		84 ± 15		83 ± 11		83 ± 13		n.s.
Satisfaction with surgery?								
Yes	No	14 (97%)	2(3%)	14(100%)	0(0%)	28 (93%)	2 (7%)	n.s.
Would you do surgery again?								
Yes	No	16 (100%)	0 (0%)	14 (100%)	0 (0%)	30 (100%)	0 (0%)	n.s. n.s.

*ACI* autologous chondral transplantation; *VAS* virtual analogue scale; *ADL* activities of daily life; *QDL* quality of daily life; *n.s.* non-significant

### Radiological outcome

**Fig. 6:**
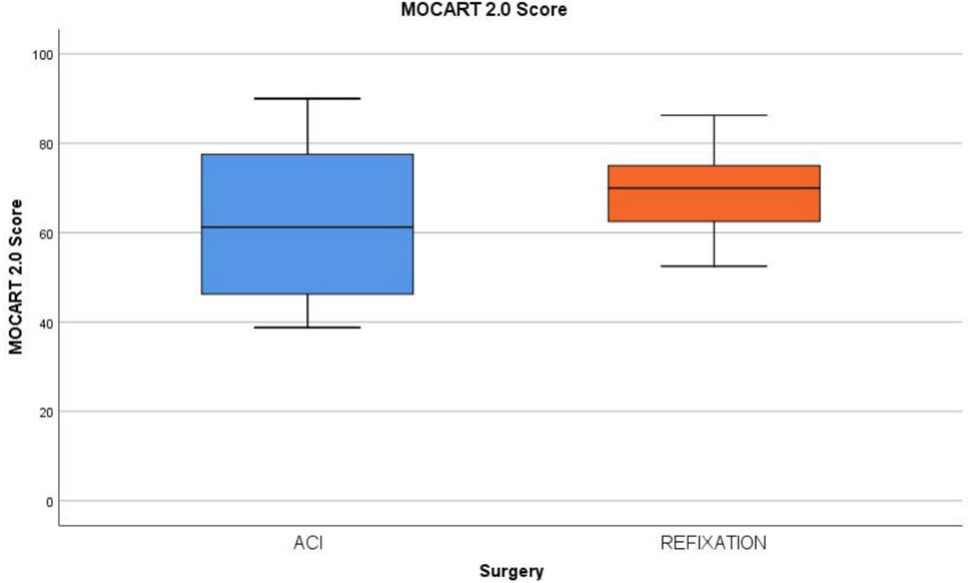
Results of MOCART 2.0 Score. The box plots show the median, the IQR and the range of the results in group comparison for the total score

At follow-up, MR images were evaluated using the MOCART 2.0 Score. There was a trend towards better results in the oFR group but the difference did not reach statistical significance (oFR: 68.2 ± 11.1; ACI: 61.1 ± 16.9; n.s.). The inter-rater reliability was excellent with an ICC of 0.847 [3] (Fig. 6). There was no significant difference regarding the MOCART 2.0 subscores (Table 3).

**Table 3:** Outcome of the MOCART 2.0 subscores of the patient cohort

	ACI	Refixation	Total	*p* value
Volume fill of Cartilage Defect	13.8 ± 4.8	16.3 ± 2.3	15.1 ± 3.8	n.s.
Integration into adjacent cartilage	11.8 ± 2.8	13.3 ± 1.3	12.6 ± 2.2	n.s.
Surface of the repair tissue	3.4 ± 2.8	4.9 ± 2.2	4.2 ± 2.6	n.s.
Structure of the repair tissue	1.9 ± 2.5	2.8 ± 2.7	2.4 ± 2.6	n.s.
Signal intensity of repair tissue	9.9 ± 2.3	10.9 ± 1.5	10.4 ± 2.0	n.s.
Bony defect or bony overgrowth	8.1 ± 1.9	7.3 ± 1.8	7.7 ± 1.9	n.s.
Subchondral changes	13.4 ± 4.3	12.5 ± 4.0	12.9 ± 4.1	n.s.

*ACI* autologous chondral transplantation; *VAS* virtual analogue scale; *ADL* activities of daily life; *QDL* quality of daily life; *n.s.* non-significant

## Discussion

The most important finding of this study was that both flake refixation and ACI following patella dislocation with (osteo-)chondral flake fractures provided excellent clinical and radiological outcomes at mid-term follow-up. In addition, there were no significant group differences in the clinical and radiological outcome scores between the groups. Regardless of the technique, the majority of the patients were able to return-to-sport and participate in activities of daily life without significant pain.

(Osteo-)chondral flake fractures present a common pathology in knee surgery. However, there is no general agreement on how to best treat this entity and the scientific evidence regarding the refixation of flake fragments in the current literature is very limited [15]. This emphasizes the need for further investigation comparing different therapeutical concepts to improve decision-making algorithms.

In the present study, refixation of the flake fragments led to good results in the VAS and the Kujala score, which indicates that most of the patients did not suffer relevant pain at follow-up. In addition, patients were able to return-to-sports and even participate in pivoting sports as shown by the results of the Tegner score and the KOOS. In addition, the majority of the patients were able to participate in activities of daily life after refixation and were not impaired by the symptoms as shown in the IKDC and KOOS. This is in accordance with recent literature that shows that the refixation, if possible, shows good clinical results [6, 10, 15]. In a recent study, Gesslein et al. showed that the clinical outcome after refixation is significantly better in comparison with the removal of the flake and debridement of the defect area. However, they did not compare the results with an alternative cartilage repair technique [6].

Besides, given the limited eligibility for refixation of (osteo-)chondral lesions, there is a need for alternative treatment methods in clinical practice. Today, commonly used techniques for cartilage regeneration in the knee are cell-based techniques like ACI. Several previous studies demonstrated that ACI provides good results for the treatment of cartilage lesions in the knee joint [2, 10, 21, 29]. However, there is also evidence that the results for patellofemoral lesions show a bigger variability and are less predictable in comparison with lesions in the femorotibial compartment [20, 22]. Interestingly, the present study showed that patients treated with ACI for flake fractures after patella dislocation show excellent results. The clinical outcome and return-to-sport rates were better than most of the results after patellofemoral ACI published so far [2, 10, 21, 29, 31]. In a current systematic review, Hinckel et al. showed that patellofemoral cartilage restoration leads to good clinical results, but with a higher complication rate in comparison to the present study [10]. The primary reason for this might be the patient selection, including only traumatic aetiology, fresh injuries, clear causality for the lesion, and young patient age.

Although there are data about the clinical results after flake refixation in the literature, there is a scarcity of data evaluating the radiological results using magnetic resonance imaging. The radiological outcome after refixation in mid-term follow-up was favourable and mostly showed good healing, as reflected by the results of the MOCART score and its subscores. The group comparison of the MR imaging results showed a trend towards worse results after ACI with higher interindividual differences, but the difference was not statistically significant. To further investigate this, a radiological study with a large patient collective should be conducted. Nevertheless, the MR imaging results after ACI were in line with existing literature on the knee joint, given the scarcity of data regarding the specific investigation of ACI on the patellofemoral joint [14, 19, 28, 29]. Siebold et al. showed a radiological success rate of 80% in a cohort of patients with MPFL reconstruction and ACI, but their collective consisted of patients with chronic instability of the patella without flake fractures [28].

Of interest, the results of the present study are of distinct clinical importance as they allow to discuss the potential treatment options with the patients to enable an informed decision-making process. An important benefit of the refixation is that it can be performed as a one-step procedure in contrast to the 2-step procedure of ACI. In addition, the refixation of the fragment allows preserving the original hyaline cartilage while ACI can form hyaline-like cartilage only [34]. Nevertheless, ACI is an excellent fallback option with similar good clinical and radiological outcome and should always be discussed with the patient before the surgery. For the surgeon, it provides a good alternative if flake refixation is not possible.

The present study has several limitations. First, the study design is retrospective and does not include preoperative data to establish a baseline regarding the joint function. However, as only patients with a primary patella dislocation were included, it can be assumed that the patient collective mostly did not have any major problems with their knee joints prior to the injury. Second, the sample size of each subgroup and the total sample size are limited and a larger study cohort may have had a stronger statistical power to detect group differences. In the present study, strict inclusion and exclusion criteria were applied to create a homogeneous study cohort with primary patella dislocations and no concomitant injuries. This strict selection process led to a limited size of the study cohort but also to a reduction of possible confounders. However, the limited group size might have affected the statistical significance of the results, with a larger group size, the statistical power would be stronger. Third, the results offer mid-term results and a conclusion regarding the development of long-term complications like arthrosis is not possible. Fourth, all patients that were eligible for refixation were treated with a refixation and were not considered for ACI, all others received an ACI. This might have had an effect on the groups. We have tested all relevant factors to ensure group comparability, nevertheless, there might have been an effect on the results of this study. To reduce this effect a randomized study with prospective design should be conducted. The time to return-to-sport was not reported. The distribution of males/females between the groups was different, the difference was not significant, nevertheless, it might have had an influence on the results [9]. Potential differences between retropatellar and trochlear pathologies were not investigated in this study. The follow-up of 81% of eligible patients is adequate, but the drop-out rate might have affected the results.

Despite these limitations, the present data demonstrated no significant difference in clinical and radiological outcomes between flake refixation and ACI, indicating that both techniques are good and reliable surgical options after retropatellar (osteo-)chondral flake fractures following primary patella dislocation. The hypothesis was discarded, as refixation of flake fractures showed no significantly better results than ACI.

## Conclusion

Open refixation of (osteo-)chondral fragments in patients after sustaining acute patella dislocation with (osteo)-chondral flake fractures led to good clinical and radiological results at a minimum follow of 24 months, showing that it is a good surgical option in the treatment algorithm. However, if open refixation is not possible ACI may be an excellent fallback option in these younger patients with equally good clinical and radiological outcomes, but requiring a second minimally invasive surgery.

## Supplementary Information

Below is the link to the electronic supplementary material.

Supplementary file1 (PDF 119 KB)
