# Supplementary material for: Excellent clinical and radiological outcomes after both open flake refixation and autologous chondrocyte implantation following acute patella dislocation and concomitant flake fractures
Source: Knee Surg Sports Traumatol Arthrosc. 2022 Feb 26;30(10):3334–42. doi: 10.1007/s00167-022-06899-3 (PMC9464151; doi:10.1007/s00167-022-06899-3)
Supplement: Supplementary file 1 — Supplementary file1 (PDF 119 KB) [file 167_2022_6899_MOESM1_ESM.pdf]

Excellent Clinical and Radiological Outcomes after both Open Flake Refixation and Autologous Chondrocyte Implantation following Acute Patella Dislocation and Concomitant Flake Fractures

Tables for online appendix

Table 4. Individual patient outcomes; Abbreviations: ACI – autologous chondral transplantation, m – male, f – female, VAS – virtual analogue scale

	**Characteristics**						**Results**				
**#**	**Group**	**Gender**	**BMI (kg/m^2)**	**Size of defect in cm^2**	**Age in years**	**Follow-up in months**	**VAS**	**Tegner**	**Kujala**	**KOOS**	**IKDC 2000**
1	ACI	m	22	3.2	18	39	7	93	89	92	61
2	ACI	f	23	1.5	17	31	7	98	98	100	59
3	ACI	f	26	6.0	16	44	5	75	60	72	78
4	ACI	f	18	2.3	25	52	3	81	87	71	81
5	ACI	f	22	4.0	16	46	7	86	86	84	81
6	ACI	f	19	1.7	18	53	4	93	91	85	
7	ACI	f	21	2.5	29	59	4	61	73	72	58
8	ACI	m	22	3.8	18	91	3	87	94	90	39
9	ACI	f	24	6.0	14	78	5	85	92	99	
10	ACI	m	22	1.5	20	33	6	82	77	82	73
11	ACI	f	19	1.5	20	29	4	78	64	59	90
12	ACI	m	28	4.0	28	60	5	91	91	89	46

13	ACI	f	19	5.0	28	39	4	93	72	78	66
14	ACI	m	24	5.0	23	68	6	86	83	92	41
15	Refixation	f	22	5.0	20	71	6	85	88	90	40
16	Refixation	m	24	4.0	22	40	7	94	88	95	69
17	Refixation	m	27	0.8	28	42	7	96	98	100	74
18	Refixation	f	23	2.3	26	44	4	72	65	64	53
19	Refixation	f	21	2.0	29	56	4	79	72	70	65
20	Refixation	f	22	1.5	16	55	6	87	87	86	59
21	Refixation	m	24	5.0	17	56	5	100	99	100	60
22	Refixation	m	22	2.0	16	64	3	83	89	87	60
23	Refixation	f	22	2.0	27	65	5	91	85	87	86
24	Refixation	f	25	1.2	14	65	5	100	93	95	73
25	Refixation	f	18	5.0	28	63	4	88	95	84	78
26	Refixation	m	27	2.0	26	97	7	83	74	77	75
27	Refixation	m	23	1.0	15	39	9	100	99	99	70
28	Refixation	m	26	3.0	23	71	3	78	80	94	86
29	Refixation	m	28	1.7	16	68	2	60	52	54	63
30	Refixation	m	26		21	27	5	66	65	57	51
